# Tolvaptan treatment for severe neonatal autosomal-dominant polycystic kidney disease

**DOI:** 10.1007/s00467-017-3584-9

**Published:** 2017-02-13

**Authors:** Rodney D. Gilbert, Hazel Evans, Kazeem Olalekan, Arvind Nagra, Mushfequr R. Haq, Mark Griffiths

**Affiliations:** 1 Regional Paediatric Nephro-Urology Unit, Southampton Children’s Hospital, Tremona Road, Southampton, SO16 6YD Hampshire UK; 2 Faculty of Medicine, University of Southampton, Southampton, UK; 3 Respiratory Unit, Southampton Children’s Hospital, Southampton, UK; 4 Pharmacy, Southampton University Hospital, Southampton, UK; 5 Radiology Department, Southampton Children’s Hospital, Southampton, UK

**Keywords:** Autosomal dominant polycystic kidney disease, Infant, Tolvaptan

## Abstract

**Background:**

Severe neonatal autosomal-dominant polycystic kidney disease (ADPKD) is rare and easily confused with recessive PKD. Managing such infants is difficult and often unsuccessful.

**Case diagnosis/treatment:**

A female infant with massive renal enlargement, respiratory compromise and hyponatraemia was treated with the arginine vasopressin receptor 2 antagonist tolvaptan. This resolved hyponatraemia, and there was no further increase in renal size.

**Conclusion:**

Tolvaptan may be a useful treatment for severe neonatal PKD.

## Introduction

Patients with severe neonatal polycystic kidney disease (PKD), whether dominant or recessive, have on occasion been treated with nephrectomy and early dialysis. This approach has a high mortality rate due in part to refractory hypotension pos-operatively due to loss of renin secretion [1] and the difficulties involved in managing completely anuric young infants. We present a patient with severe neonatal autosomal-dominant PKD (ADPKD) due to biallelic inheritance of *PKD1* mutations. She was successfully treated with tolvaptan, a selective, competitive inhibitor of the vasopressin V2 receptor.

## Case report

**Fig. 1:**
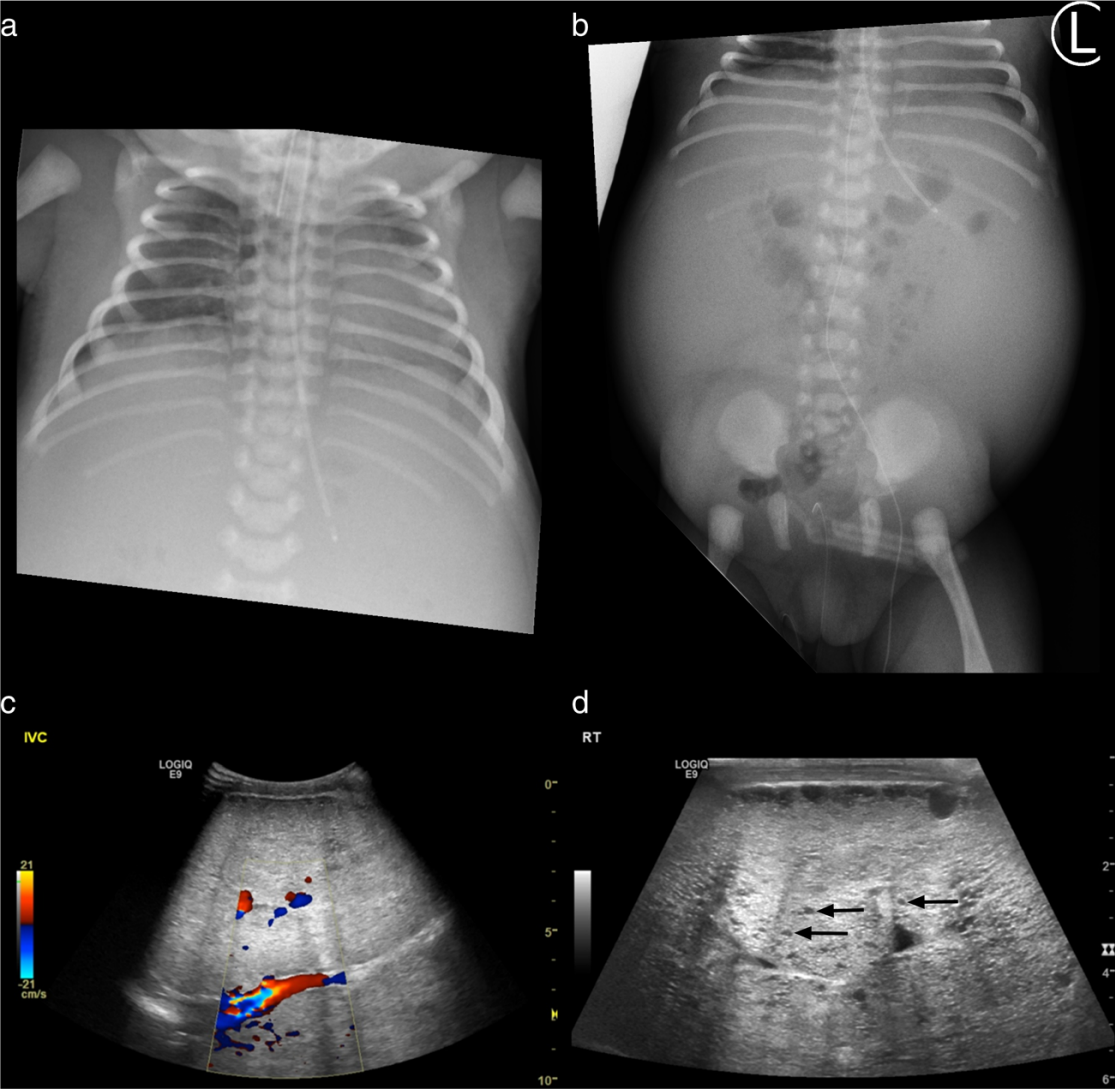
**a** Chest radiograph showing distended abdomen and small chest. **b** Abdominal radiograph showing distended abdomen with bilateral soft tissue masses and central bowel gas, also seen within a right inguinal hernia. **c** Abdominal ultrasound scan showing extremely enlarged and echobright kidneys with reduced corticomedullary differentiation compressing the inferior vena cava. The right kidney measured 10.2 cm long and the left 10.7 cm. The depth of both kidneys was 5.6 cm and transverse dimension 7.5 cm. At term, mean sonographic renal length was 4.48 ± 0.31 cm. **d** High-resolution view showing multiple small cysts, three of which are indicated by *arrows*

A 22-year-old woman with a well-established diagnosis and family history of ADPKD was referred for antenatal counselling after a scan at 17 weeks showed abnormal kidneys in the fetus. Serial antenatal ultrasound scans confirmed large, echogenic kidneys with no resolvable cysts. Urine was seen within the bladder, but the amniotic fluid index was consistently at the lower end of the normal range. The female infant was delivered by elective caesarean section at 37 weeks’ gestation weighing 3.370 kg. She was initially pink with good tone, but as soon as the cord was clamped, she became cyanosed and floppy, showing no respiratory effort and heart rate that dropped to 60/min. Inflation breaths administered via a face mask produced no visible chest movement and she was intubated. Ventilation pressures of 35/5 cm water and an inspired oxygen concentration of 50% were required to keep arterial oxygen saturation >90%. Further examination revealed a relatively small chest (Fig. 1a) and a distended abdomen, with massively enlarged kidneys (Fig. 1b). There was a left inguinal hernia; female genitalia and the rest of the examination were normal.

*PKD1* sequencing showed a heterozygous c.7483 T > C; p.(Cys2495Arg), highly likely pathogenic [2], missense mutation in exon 18 and the hypomorphic variant [3] c.9829C > T; p.(Cys 2495Arg) in exon 29. The former was shown to be inherited from her mother and the latter from her father. Urine output in the first 24 h was 0.2 ml/kg/h. Plasma creatinine rose to a maximum of 189 μmol/L on day 4 and gradually reduced thereafter. Plasma sodium fell to a minimum of 126 mmol/L on day 3 despite fluid restriction to 20 ml/kg per day, which was mostly given IV. Sodium administration was 1.5 mmol/kg per day, with feeds of maternal breast milk 2 ml every 2 h. Systolic blood pressure was in the mid-80s. She was successfully extubated and managed with face-mask continuous positive airway pressure (CPAP) on day 3.

At 7 days of age, she was started on parenteral nutrition due to intolerance of enteral feeds of adequate volume. By day 8, she was weaned off oxygen and by day 9 was tolerating sufficient enteral feed to stop parenteral nutrition. She had oedema of the legs and labia. On day 14 she became hypertensive, with systolic blood pressures ranging from 98 to 106 mmHg. Treatment with captopril was started. She developed respiratory acidosis and required high-flow humidified oxygen. A sleep study on day 16 confirmed frequent arterial oxygen desaturations and hypercapnoea, with a mean pCO_2_ of 8.03 kPa. She was given supplementary oxygen when sleeping.

Facial and leg oedema became increasingly problematic and was associated with hyponatraemia. The patient had continued respiratory distress with flaring of the alae nasi, grunting, and opisthotonic posturing. An abdominal ultrasound showed that the enlarged echogenic kidneys with multiple small cysts were compressing the inferior vena cava, and oedema was attributed to obstructed venous return (Fig. 1c, d). Hyponatraemia was attributed to nonosmotic stimulation of vasopressin release due to activation of the thoracic baroreceptors caused by impaired cardiac filling, exacerbated by reduced free water clearance due to impaired renal function. The oedema was initially managed with furosemide, but this aggravated hyponatraemia and caused hypokalaemia. Approval for treatment with tolvaptan was therefore sought to combat hyponatraemia and prevent further complications by minimising renal growth. While waiting for approval, hyponatraemia was managed with sodium chloride supplements, which caused her systolic blood pressure to rise to >110 mmHg.

Consent for treatment for an unlicensed and unexplored indication was given by the parents. Tolvaptan was started in at 0.15 mg/kg per day on day 31 of postnatal life, and the sodium supplements were stopped at the same time. Urine output increased from 1.75 ml/kg per hour before tolvaptan to 2.4 ml/kg per the day of the first dose. Plasma sodium initially remained constant at 136 mmol/L, but 3 days later, the patient became mildly hyponatraemic (plasma sodium 131, falling to nadir of 129 on day 5 of tolvaptan therapy). This was attributed to variable dosing. There is no licensed liquid formulation of tolvaptan, so initially, the dose was administered by crushing a tablet and suspending it in water. However, because tolvaptan is insoluble in water (0.00005 w/v% at 25 °C) [4] and there were concerns about accuracy of the administered dose, a suspension was made by the pharmacy as follows: tolvaptan tablets (Samsca^®^) 4 × 15 mg tablets, Oral-Plus^®^ 30 ml, Oral-Sweet^®^ to 60 ml, giving a final strength of 1 mg/ml. The expiry date was set at 7 days. Thereafter, plasma sodium concentration was consistently within the normal range.

Because of concerns of hypernatraemia, the tolvaptan dose was slowly increased while increasing the feed volume. By 3 months of age, she was administered 0.5 mg/kg per day as a single dose. At 6 months, the dose was increased to 0.7 mg/kg per in two divided doses, with two thirds given in the morning and one third late afternoon. At 9 months of age, the dose was increased to 1 mg/kg per day. On this dose, her urine osmolality has been consistently <300 mOsm/kg.

On the day of the first tolvaptan dose, plasma creatinine concentration was 103 μmol/L. After 1 month of tolvaptan therapy, it had fallen to 77 μmol/L and at 12 months 43 μmol/L, giving an estimated glomerular filtration rate (eGFR) of 55 ml/min/1.73 m^2^. This remained similar at 18 months of age, when plasma creatinine was 41 μmol/l and eGFR 60 ml/min/1.73 m^2^. Kidney size (assessed by ultrasound) has not changed since starting tolvaptan. While there is no evidence of a reduction in renal size, a 3% volume change would be within the limits of accuracy of the technique. Breathing is normal, and oxygen administration was stopped following a normal sleep study at 12 months of age. Development is normal.

## Discussion

ADPKD is the most common monogenic disease causing end-stage renal failure, occurring in 1:400 to 1:1000 individuals worldwide [5]. Although ADPKD is dominant, with almost 100% penetrance at the individual level, it appears to be recessive at the cellular level. Only a relatively small proportion of nephrons develop cysts. Several investigators demonstrated deletion or other inactivating somatic mutations in the remaining normal *PKD1* or *PKD2* gene in epithelial cells lining individual cysts [6]. In further support of a gene–dosage effect, several authors reported unusually early and severe disease, often mimicking renal manifestations of recessive PKD, in patients with either biallelic abnormalities of *PKD1* or *PKD2*, such as our patient [1, 7, 8], or a pathogenic mutation of *PKD1* or *PKD2* in association with a mutation in another gene associated with renal cystic disease, such as *PKHD1* or *HNF1β* [9].

Reduced expression of polycystin 1 or 2 results in accumulation of cyclic adenosine monophosphate (cAMP) within the cytoplasm of renal tubular cells [10]. Although under normal circumstances cAMP inhibits cell proliferation, in ADPKD it increases both cell proliferation and fluid secretion [11]. Arginine vasopressin (AVP) is the main agonist of adenyl cyclase in collecting ducts, and patients and experimental animals with PKD have increased levels of circulating AVP and upregulation of AVP- and cAMP-dependent genes, such as the V2 receptor and aquaporin-2. In adult patients with ADPKD, tolvaptan slowed the increase in kidney size and the rate of glomerular filtration rate (GFR) decline [12], with an average reduction in kidney volume (assessed by MRI) of 3.1% after 1 week of treatment [13]. Tolvaptan has now been licensed for treating adults with ADPKD in a number of jurisdictions.

The rationale for treating this patient was to treat the hyponatraemia and presumed excessive AVP secretion (plasma copeptin was not measured) and secondly to prevent or reduce renal growth as much as possible. Kidney volume tends to increase at a greater rate in children than in adults [14], and those with higher AVP secretion have greater disease severity [15]. Our patient was therefore considered to be at high risk of a rapid increase in renal size. On tolvaptan therapy, she has grown well and maintained a normal plasma sodium concentration, while her kidneys have not grown and she is now completely untroubled by renal size. Estimated GFR also improved. While it is impossible to know whether it would have improved to the same extent had the patient survived without tolvaptan therapy, renal function may even have deteriorated. Polyuria has not been a significant problem, and there have been no incidents of hypernatraemia or hepatotoxicity. Although we cannot know what renal growth without tolvaptan would have been, we suggest that this treatment was beneficial in our patient. Further study is necessary to determine the role of tolvaptan in managing infants with severe dominant or recessive PKD.
